# Supplementary material for: Safety Climate and Occupational Injuries in the Iron and Steel Industries in Tanzania
Source: Int J Environ Res Public Health. 2025 Aug 31;22(9):1372. doi: 10.3390/ijerph22091372 (PMC12470045; doi:10.3390/ijerph22091372)
Supplement: Supplementary file 1 [file ijerph-22-01372-s001.zip › ijerph-3776133-supplementary.pdf]

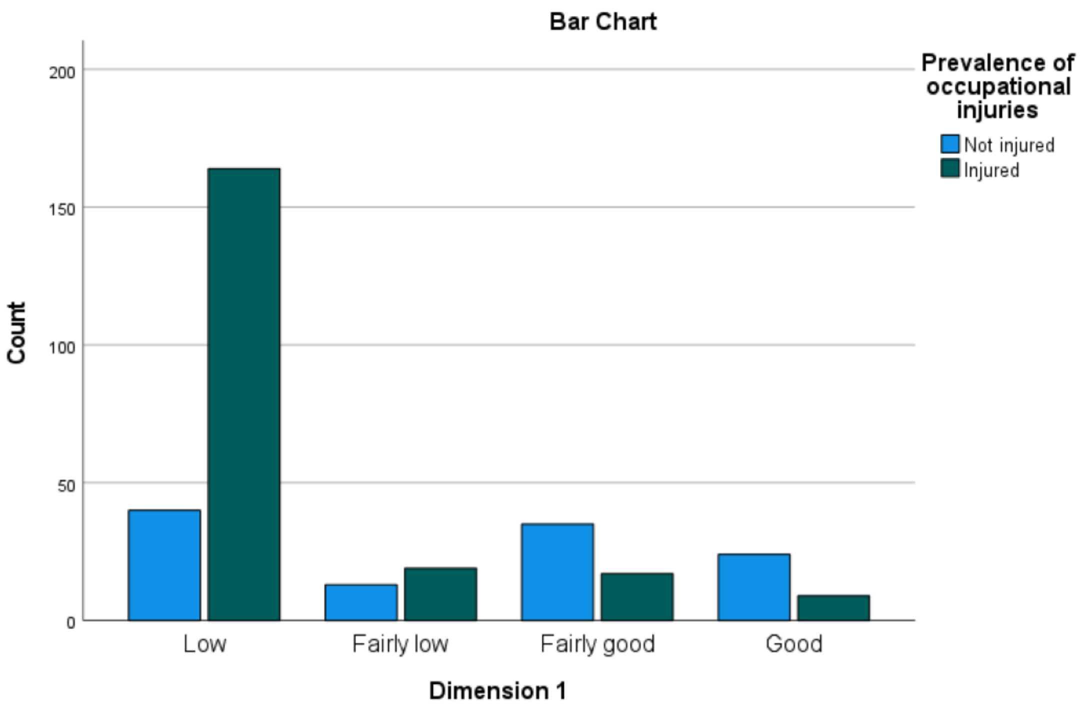

Figure S1. Safety climate scores(Management safety priority and ability) and occupational injuries among workers in the iron and steel industry.

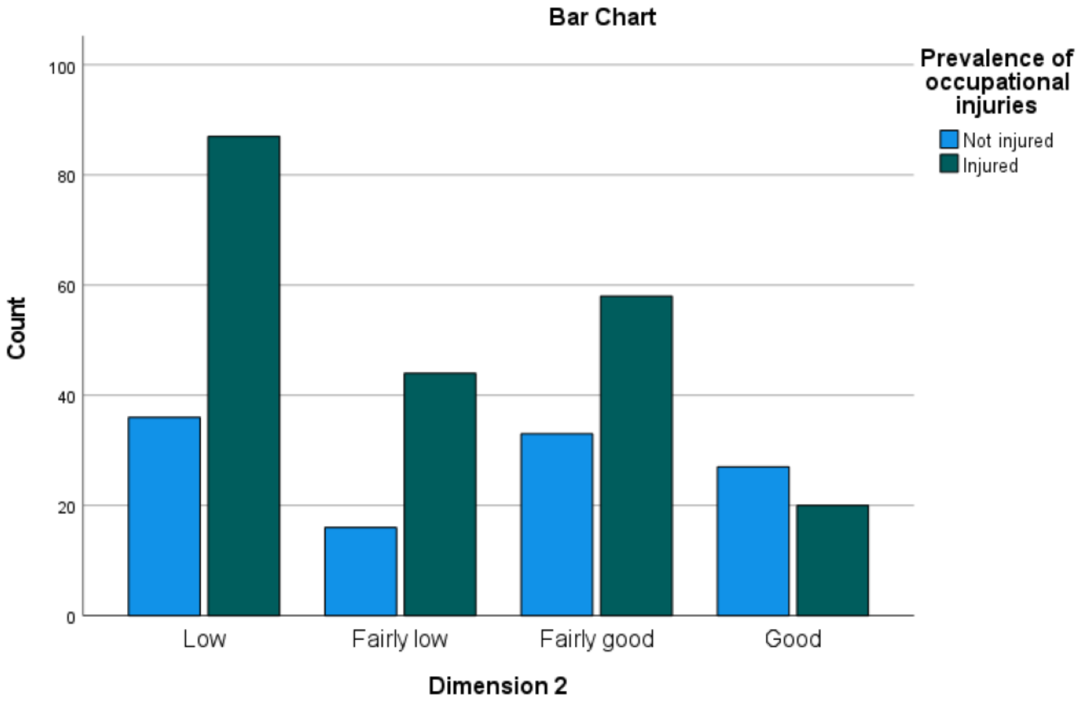

Figure S2. Safety climate scores (Management safety empowerment) and occupational injuries among workers in the iron and steel industry.

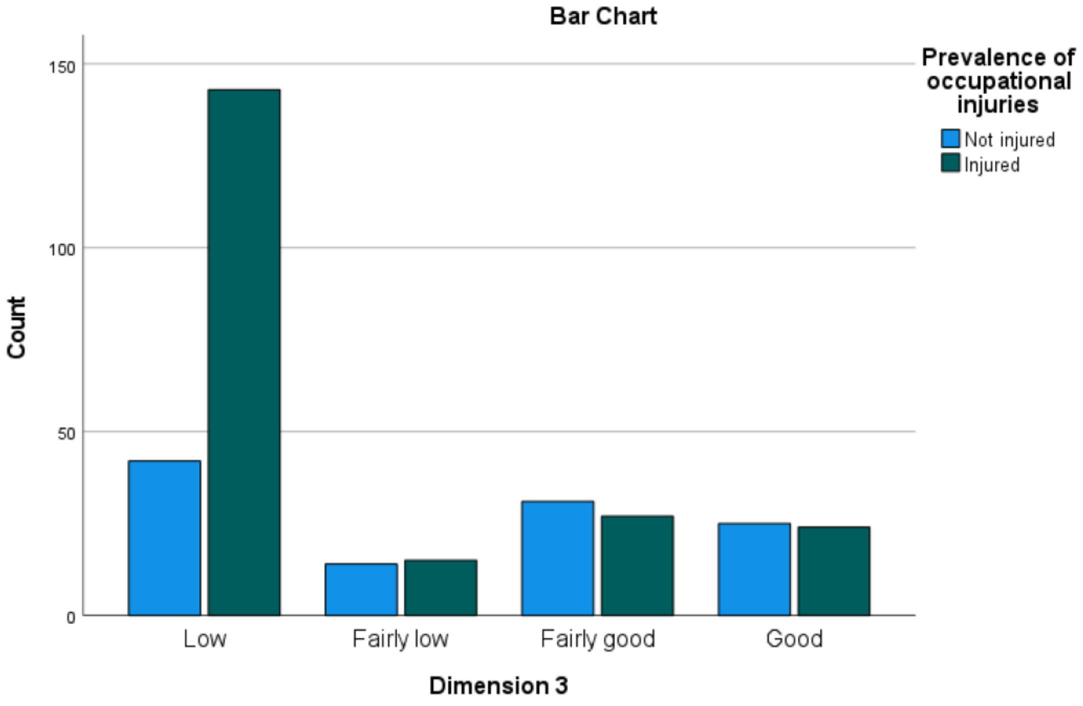

Figure S3. Safety climate scores (Management safety justice) and occupational injuries among workers in the iron and steel industry.

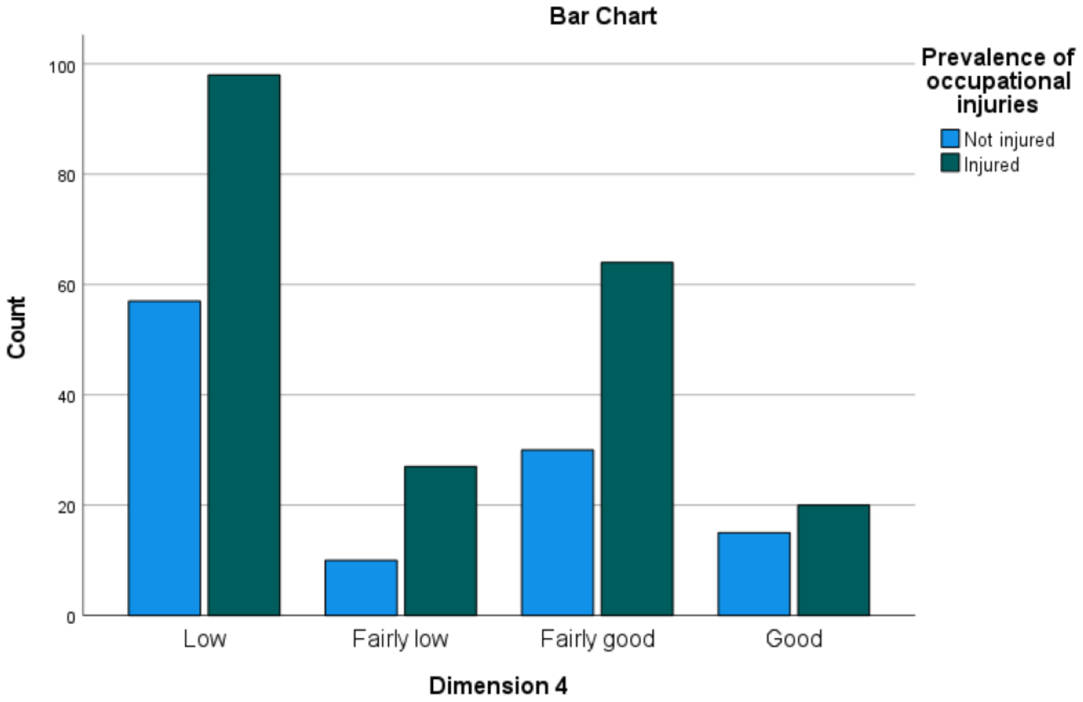

Figure S4. Safety climate scores (Worker safety commitment) and occupational injuries among workers in the iron and steel industry.

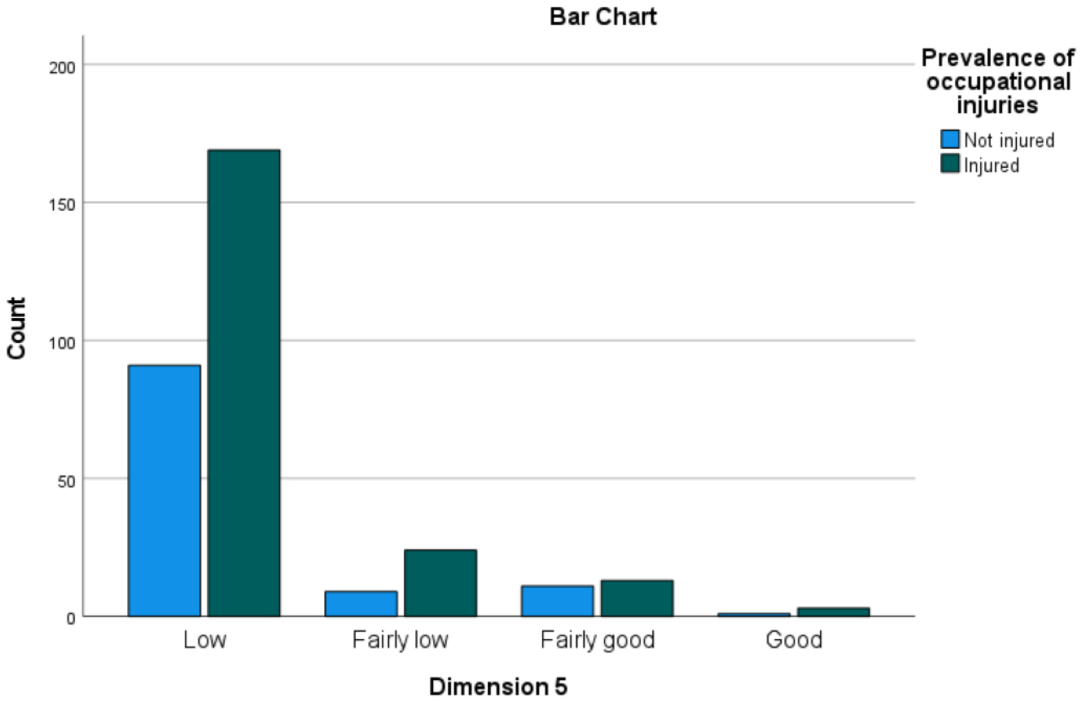

Figure S5. Safety climate scores (Workers' safety priority and risk non-acceptance) and occupational injuries among workers in the iron and steel industry.
